# Sex Differences in Language Across Early Childhood: Family Socioeconomic Status does not Impact Boys and Girls Equally

**DOI:** 10.3389/fpsyg.2015.01874

**Published:** 2015-12-02

**Authors:** Stéphanie Barbu, Aurélie Nardy, Jean-Pierre Chevrot, Bahia Guellaï, Ludivine Glas, Jacques Juhel, Alban Lemasson

**Affiliations:** ^1^EthoS- Animal and Human Ethology, UMR 6552, University of Rennes 1 - CNRSRennes, France; ^2^LIDILEM - Linguistique et Didactique des Langues Etrangères et Maternelles, University of Grenoble AlpesGrenoble, France; ^3^IUF - Institut Universitaire de FranceParis, France; ^4^LECD - Laboratory of Ethology, Cognition, Development, University of Paris Ouest Nanterre La DéfenseNanterre, France; ^5^Center for Research in Psychology, Cognition, Communication, University of Rennes 2Rennes, France

**Keywords:** language acquisition, gender, SES, phonological development, French liaison, preschoolers

## Abstract

Child sex and family socioeconomic status (SES) have been repeatedly identified as a source of inter-individual variation in language development; yet their interactions have rarely been explored. While sex differences are the focus of a renewed interest concerning emerging language skills, data remain scarce and are not consistent across preschool years. The questions of whether family SES impacts boys and girls equally, as well as of the consistency of these differences throughout early childhood, remain open. We evaluated consistency of sex differences across SES and age by focusing on how children (*N* = 262), from 2;6 to 6;4 years old, from two contrasting social backgrounds, acquire a frequent phonological alternation in French – the liaison. By using a picture naming task eliciting the production of obligatory liaisons, we found evidence of sex differences over the preschool years in low-SES children, but not between high-SES boys and girls whose performances were very similar. Low-SES boys’ performances were the poorest whereas low-SES girls’ performances were intermediate, that is, lower than those of high-SES children of both sexes but higher than those of low-SES boys. Although all children’s mastery of obligatory liaisons progressed with age, our findings showed a significant impeding effect of low-SES, especially for boys.

## Introduction

Language is one of mankind’s key abilities and a universal feature of human development; primary emphasis has therefore often been placed on documenting universal processes of language acquisition by focusing on children’s achievement of milestones, relegating inter-individual variation to the background. However, time courses of language acquisition vary greatly among children. These variations, far from being an awkward background noise, are relevant for understanding the mechanisms that underpin language acquisition by providing a window onto the correlates and causes of language development (Bates et al., 1995). Family socioeconomic status (SES) and child sex have been repeatedly pointed to as sources of inter-individual variation in language development. While studies on family SES have rovided consistent findings about the detrimental effect of lower-SES in various language skills, this has not been the case for studies looking at the sex of the child. Despite widely held beliefs about sex differences in language development, with the prevalent stereotype being that boys lag behind girls, empirical evidence is mixed. There are also inherent problems in interpreting published data on child sex effect. These derive from the variety of study designs, the different language domains examined, and the heterogeneous populations studied (in terms of participants’ ages and SES, in particular) which make interpretation and comparison extremely difficult. Whether child sex is a meaningful source of variation in language abilities has thus remained a matter of debate across the decades, considered important by some (Maccoby and Jacklin, 1974; Fenson et al., 1994), but negligible by others (Hyde and Linn, 1988; Hyde, 2005; Wallentin, 2009); discrepancies between studies and inconsistent findings undoubtedly fuel the continuing debate. While family SES and child sex have both been the focus of a great deal of research, less attention has been directed toward understanding how these factors interact across ages in order to account for between-child differences in language development. This knowledge is important in order to go beyond the current debate on the mere existence of sex-related differences in language and to improve our understanding of the detrimental effect of lower-SES in relation to child sex. In this perspective, the aim of the present study was to investigate whether sex differences are consistent across socioeconomic subgroups and whether family SES impacts children of both sexes equally, by comparing children’s language skills at the two extremities of the socioeconomic strata across a wide age range covering the preschool years.

Family SES has been repeatedly identified as a highly significant predictor of language development (Hoff, 2003; Huttenlocher et al., 2010; Rowe, 2012). SES differences, beginning within the second year of life (Fenson et al., 1994; Fernald et al., 2013) and widening over time (Huttenlocher et al., 2010; Zambrana et al., 2012; Fernald et al., 2013), have been found for vocabulary and syntax skills in both production and comprehension across preschool years (Huttenlocher et al., 2002; Fish and Pinkerman, 2003; Le Normand et al., 2008; Zhang et al., 2008) and school years (Hart and Risley, 2003; Reynolds and Fish, 2010). As lower SES is associated with poorer language outcomes, low-SES is seen as a risk factor impairing early language development and delaying later school achievement. Given these developmental outcomes, information concerning consistency of sex differences across the socioeconomic strata and how environmental experiences affect children in relation to their sex is an important issue.

With regard to sex differences in language development, a growing number of recent studies report a consistent girl advantage across the first 30 months of life for various aspects of language, from early communicative gestures (Özçalışkan and Goldin-Meadow, 2010) to early vocabulary growth (Huttenlocher et al., 1991; Bauer et al., 2002), morphosyntactic growth (Hadley et al., 2011), and vocabulary size and syntactic complexity (Fenson et al., 1994; Galsworthy et al., 2000; Lutchmaya et al., 2002b; Van Hulle et al., 2004; Berglund et al., 2005; Kern, 2007; Westerlund and Lagerberg, 2008; Bouchard et al., 2009; Lovas, 2011; Simonsen et al., 2014). These early differences are evidenced across a wide-range of languages, countries, and ecological settings (Bornstein and Cote, 2005; Eriksson et al., 2012: in 10 European language communities including French) and mostly in mixed-SES samples. However, these early differences are not systematically found across studies in all language skills; better performances by girls have been reported more consistently for vocabulary production. The girls’ advantage is also likely to be small, with child sex explaining only a small amount of the variance (Fenson et al., 1994; Galsworthy et al., 2000; Berglund et al., 2005; Reilly et al., 2007). When considering the real-life consequences of sex stereotypes about language, this suggests that the actual sex difference is not sufficiently large to justify the widespread belief that late development of language in a boy is no cause for concern, as evidenced by physician referral practices for children with developmental delay (Sices et al., 2004). Nevertheless, these studies show that when a sex difference is found, it is always in the same direction with girls acquiring language more rapidly than boys during the first years of life.

Evidence remains scarce and is less consistent over the preschool years. First of all, there are fewer studies of preschool years compared to the ever growing number of studies focusing on the early years of language development, thanks to the international adaptation of parental inventories in various languages and countries. Using various design and language measures in mixed-SES samples, these studies have reported sex differences up to 36 months for overall language comprehension (Zambrana et al., 2012) and lexical and grammatical skills (Zhang et al., 2008), but not after that age (Le Normand et al., 2008; Farrant et al., 2013), as boys seem to catch girls up when approaching 3 years old (Simonsen et al., 2014). Longitudinal surveys show a girls’ advance in vocabulary growth during the rapid period of acceleration from 14 to 26 months of age (Huttenlocher et al., 1991), but not later on during the preschool years whether in production (Huttenlocher et al., 2010: from 14 to 46 months) or in comprehension (Rowe, 2012: from 30 to 54 months). On the other hand, in studies focusing on middle-SES families, a small but consistent advantage has been found for girls for almost all language measures assessed between 2 and 6 years old, but not before or after these ages (Bornstein et al., 2004). Therefore, sex differences have not consistently been found throughout childhood. Whether studies found sex differences or not seems at least in part to depend on children’s ages and family SES.

A few studies have, however, considered the impact of child sex and SES concurrently in order to assess which demographic factors are significant predictors of language development and how they contribute to individual variation across ages. When considering large cohorts of young children in mixed-SES samples, studies have shown that during the first 2 years of life, child sex is the most influential factor whereas family SES does not contribute significantly to vocabulary production (Fenson et al., 1994; Berglund et al., 2005; Reilly et al., 2007; Westerlund and Lagerberg, 2008). Around 3 years of age, both child sex and family SES make a small but significant contribution to vocabulary skills, but not systematically to syntactic skills, with girls and high-SES children having better performances (Fenson et al., 1994; Zhang et al., 2008). At later ages during the preschool years, family SES become a strong predictor of vocabulary and syntactic growth, contrary to child sex that is no longer significant (Huttenlocher et al., 2010; Rowe, 2012). Therefore, the relative influence of demographic factors on language changes with age across childhood; the association between SES and vocabulary gets stronger over the course of time (Fenson et al., 1994; Hoff-Ginsberg, 1998) while the influence of child sex seems to attenuate after 3 years of age.

By focusing on low-income populations, some studies nevertheless suggest that the girls’ advance may be consistent across early childhood and beyond, at least for children from lower-SES. Girls from low-income populations showed better vocabulary and syntactic skills than boys in spontaneous speech in the first years of life (Morisset et al., 1995). They also outperformed boys in standardized evaluations from kindergarten to middle childhood (Locke et al., 2002; Fish and Pinkerman, 2003; Reynolds and Fish, 2010). The inconsistency of sex differences in the literature thus also calls into questions the representativeness of samples in studies based on mixed-SES samples or collapsed demographic groups. As noted repeatedly (Fenson et al., 1994; Simonsen et al., 2014), families at the low end of the socioeconomic scale are quite under-sampled even in large cohorts of children as attrition is higher in low-SES. It is therefore important to consider possible interaction between child sex and family SES.

While a few studies have simultaneously assessed the contribution of child sex and family SES to language development, studies looking at how these factors interact are even more rare. One longitudinal survey has investigated overall language comprehension between 18 and 36 months (using a short maternal report composed of five items for 18 months and seven items for 36 months) in a large cohort of diverse SES (Zambrana et al., 2012). The authors show that sex differences increase with decreasing level of maternal education (one of the common indices used to reflect family SES along with parental occupations and incomes) and that maternal education has a greater impact on change in language comprehension across ages in boys than in girls. However, such interaction has not been reported for other language skills, whether in younger children (vocabulary production and comprehension at 18 months: Berglund et al., 2005) or older children (lexical productivity and diversity between 24 and 48 months: Le Normand et al., 2008). Therefore, whether sex differences are consistent across the socioeconomic strata after age 3 and how SES impacts child language in relation to their sex across early childhood remain to be understood.

To address these issues, we focused on how young children acquire a frequent phonological alternation in French: the liaison. Phonological development remains poorly explored with regard to individual differences related to children’s sex and SES compared to the extensive literature devoted to vocabulary and syntax. A liaison consists of the production of a consonant between two words (word1 and word2) in fluent speech (e.g., [z] in *les ours* [lezurs] ‘the bears’) when the first word ends with a consonant – not produced when pronounced in isolation – and the second word starts with a vowel. Word1 determines the possibility of producing a liaison and its phonetic nature. For example, the word1 *un* (‘a’/‘one’) triggers a liaison with the consonant /n/, the word1 *deux* (‘two’) triggers the liaison with the consonant /z/ and the word1 *petit* (‘little’) triggers the liaison with the consonant /t/ whereas *joli* (‘pretty’) does not trigger any liaison when it is a word1. Liaisons – with consonants /n/, /z/, and /t/ in 99.7% of the cases – are frequent in adult speech as a liaison context occurs on average every 16 words (Boë and Tubach, 1992). They represent a challenging task for young learners in word segmentation as children have to extract words from the flow of speech when word and syllable boundaries differ, causing young children to make frequent errors (e.g., *les [n]ours* instead of *les [z]ours* “the bears”) (Chevrot et al., 2009). Liaisons have heuristic value as early word segmentation abilities are related to later language development (Junge et al., 2012; Singh et al., 2012). They are also a strong indicator of the frequency effect (i.e., liaisons occur to a greater extent in high-frequency word combinations than in low-frequency combinations; Bybee, 2001; Dugua et al., 2009) and are thus good candidates for exploring the influence of the input to which children are exposed.

We focused here on the acquisition of obligatory liaisons, namely liaisons that are systematically realized by adult speakers (i.e., 100% production rate) whatever their sociodemographic characteristics and the situational context of speech. Liaisons are obligatory in only four linguistic contexts: after preverbal clitics (*ils arrivent* [ilza
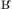
iv] ‘they come’), after determiners (*un arbre* [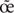
na*R*b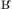] ‘a tree’), in verb + clitic inversion (*Comment dit-on?* [kom
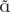
dit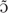] ‘how do we say?’), and in some frozen expressions (*tout-à-fait* [tutafε] ‘absolutely’; Durand and Lyche, 2008). Liaison acquisition is not easy: it takes approximately 6 years for French children to fully master obligatory liaisons (Chevrot et al., 2007, 2009; Dugua et al., 2009 for a detailed presentation of a usage-based model of their acquisition and related experimental and corpus-based studies). Liaison acquisition is not restricted to phonological abilities as its functioning involves different linguistic levels: phonology, lexicon, morphology, and syntax (Chevrot et al., 2005). Notably, authors have previously shown that the acquisition of prenominal liaisons (i.e., in the context determiner + noun) involves interactions between various levels of linguistic knowledge: learning of the phonological alternation, segmentation, and stabilization of the phonological representation of new words, and grammatical organization of the nominal phrase (Chevrot et al., 2009; Dugua et al., 2009). Investigations of the acquisition of liaisons therefore address basic issues in various domains of language development. With regard to inter-individual variations in the acquisition of obligatory liaisons, family SES has been shown to impact both their production and evaluation across preschool years, with high-SES children outperforming low-SES children (Chevrot et al., 2011; Barbu et al., 2013); however, sex differences have never been studied and nor have their interactions with family SES. To this end, we investigated rate of liaison acquisition by boys and girls from two contrasting social backgrounds (high- versus low-SES) across preschool years (age range = 2;6–6;4 years) by using a picture naming task eliciting the production of obligatory liaisons in determiner + noun sequences.

## Materials and Methods

### Participants

The participants were 262 children (129 boys and 133 girls) recruited primarily from local preschools, kindergartens, and surrounding communities in various cities of France (Paris, Rennes, Grenoble, Nîmes and surrounding areas). Informed consent was obtained from parents as well as from schools and nurseries when the research took place on their premises. Children were also asked to give their assent and were not tested if they did not want to participate. The University of Grenoble Research Ethics Committee approved the protocol and procedure (CERNI 2012-09-18-05). Parents of participating children filled out a questionnaire concerning children’s demographic information. The selection criteria were that children were from (1) monolingual French-speaking homes and (2) two contrasting socioeconomic backgrounds (high-SES versus low-SES). Family SES was based on both parents’ occupations following the nomenclature of the French National Institute of Statistic and Economic Studies (INSEE, 2003): high-SES parents belonged to group 3 (e.g., teachers and scientific professions, senior managers, engineers) and low-SES parents to group 6 (e.g., industrial, artisanal and agricultural workers and drivers). Both parents were from the same SES. When one of the parents was unemployed (i.e., did not work outside the household), only the occupation of the working parent was taken into consideration.

Following a cross-sectional design, the children, from 2;6 to 6;4 years old (Mean age = 51.08 months, *SD* = 12.56), were selected so as to constitute 4 age groups, balanced according to sex and SES, corresponding to French nursery school grades: 2–3, 3–4, 4–5, and 5–6 years old (see **Table 1** for demographics). All the children attended nursery school, except those in the youngest age group (69% of them did, with similar proportions of school/out-of-school children in sex × SES groups, *df* = 3, χ^2^ = 1.43). Indeed, less than 20% of the 2-year-olds attend school in France whereas nearly all children do so when they are 3 years old (French Ministry of Education, 2010). Most of the out-of-school children attended kindergarten (daycare type was not reported by the parents for five children). A 4 (age) × 2 (sex) × 2 (SES) ANOVA confirmed that age differed significantly between age groups [*F*(3,246) = 1619.22, *p* < 0.0001] but not within each age group whatever their sex or SES (all *p* > 0.20).

**Table 1:** Age, sex, and socioeconomic status (SES) composition of the study sample.

	2–3 years			3–4 years			4–5 years			5–6 years		
	*M*	*SD*	*n*	*M*	*SD*	*n*	*M*	*SD*	*n*	*M*	*SD*	*n*
High-SES												
Girls	35.5	2.2	16	43.6	2.6	15	55.3	3.1	19	67.4	3.3	18
Boys	34.6	2.5	18	44.1	2.3	16	56.3	2.8	15	67.7	2.9	19
Low-SES												
Girls	35.1	1.5	15	43.6	2.8	17	54.6	3.3	17	67.1	3.5	16
Boys	34.6	2.3	13	44.8	2.8	14	55.7	2.9	18	67.8	3.3	16
Overall	35.0	2.2	62	44.0	2.6	62	55.5	3.1	69	67.5	3.2	69

*M, Mean age in months; SD, standard deviation; *n*, number of children.*

### Materials and Procedure

We used a picture naming task to elicit the production of liaisons by children. Therefore, children were asked to produce word1-word2 sequences related to pictures of animals and objects that represented the six selected vowel-initial word2s, i.e., six nouns starting with a vowel: *ours, arbre, avion, escargot, éléphant, ordinateur* (*‘bear, tree, plane, snail, elephant, computer’*). The selection criteria were (1) that 2–6-year-olds accurately named these familiar objects in picture tasks (Cannard et al., 2006) and (2) the syllabic length of words, with the same number of short (*ours, arbre, avion*) and long words (*escargot, éléphant, ordinateur*) as children’s liaison errors increase with the syllabic length of word2s (Wauquier-Gravelines, 2005). To elicit the production of obligatory liaisons, word1s were two determiners: *un* (‘a/one’) and *deux* (‘two’) that trigger, respectively, the frequent liaison consonants /n/ and /z/. Therefore each animal or object was presented in one or two exemplars. The pictures were taken from children’s picture books and presented on cards. Twelve target sequences contained an obligatory liaison.

Target sequences with vowel-initial word2s alternated with sequences containing word2s starting with a consonant (i.e., not inducing a liaison) so as to avoid interference between liaison consonants. The six consonant-initial word2s were: *lit, singe, ballon, balai, cochon, camion* (*‘bed, monkey, ball, broom, pig, truck’*). Therefore, the children produced 24 word1-word2 sequences during the task: 12 sequences “determiner + vowel-initial word2” and 12 sequences “determiner + consonant-initial word2”. The order of sequences was pseudo-randomized for each child, although the alternation between target and distractor sequences was maintained.

The task was conducted individually at school for most of the children (64.9%), at home (34.3%) or occasionally at kindergarten (0.8%). At the beginning of the task, the experimenter told the child “I am going to show you pictures and you will tell me what there is on the picture.” To ensure that children understood the instructions, the experimenter started with an example with a consonant-initial word2. During the task, children’s productions were audio-taped for later transcription.

### Coding and Reliability

A liaison was considered to be correctly realized when a child produced the appropriate liaison consonant (e.g., /z/ in *deux ours* [døzu
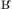
s] ‘two bears’). Children can make two types of liaison errors: replacements when the sequence is produced with an inappropriate liaison consonant (e.g., [dønu
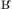
s]) and omissions when the sequence is produced without a liaison (e.g., [døu
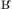
s]). We also recorded atypical responses when a child named a wrong word2 (e.g., *mammouth* ‘mammoth’ instead of ‘elephant’) or dropped the initial vowel of word2 (e.g., [dølef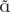] instead of *deux éléphants* ‘two elephants’) making the liaison impossible. Non-responses were noted when children remained silent. Three trained coders did the transcriptions with high reliability (Cohen’s kappa = 1 for each coded category). Inter-coder reliability was established from data for 25 subjects selected randomly (that is 300 liaison occurrences).

### Measures and Statistical Analyses

The analyses were carried out on children’s effective responses, that is, on correct word1-word2 sequences that allowed the production of a liaison (i.e., atypical responses and non-responses, which represented 14% of the sequences presented to the children, were removed from the analyses). The analyses were thus computed on a total of 2691 data points (for each age group, 2–3 years: 501, 3–4 years: 609, 4–5: 770, 5–6 years: 811 contexts of liaison).

All the modalities of children’s effective responses were included in the analyses with the correct (i.e., liaisons correctly produced) versus incorrect responses (i.e., replacements and omissions) as the dependent variable using binomial mixed logit models (for a detailed review of categorical data analyses, see Jaeger, 2008). Generalized Linear Mixed Models (GLMM) were performed using R Software (R Development Core Team, 2015) with the lme4 package (Bates et al., 2015). The children’s age (four age groups: 2–3, 3–4, 4–5, 5–6 years), sex (male, female) and SES (high, low) were included in the analyses as fixed factors so as to assess their effects and interactions on the children’s productions of correct liaisons. The children and the items of the picture naming task were included as random factors. The best fitting model was selected by comparing successively the competing models. In the results section, we provided the Akaike Information Criterion (AIC) and the Bayes Information Criterion (BIC) for each model and the Likelihood ratio test for model comparisons. *Post hoc* multiple comparisons tests were performed using Tukey contrasts (multcomp package, Hothorn et al., 2008). All results reported as significant are at an alpha level of *p* < 0.05.

## Results

First, the implementation of subjects and items as random factors significantly improved the likelihood of the full model including all the fixed effects and their interactions (model 1 with all fixed factors and interactions without random factors: AIC = 2454.8, BIC = 2549.2; full model 2 with subjects as random factors: AIC = 2317.1, BIC = 2417.3, model 2 vs. model 1: Likelihood ratio test, *LR* = 503.61, *df* = 1, *p* < 0.000; full model 3 with both subjects and items as random factors: AIC = 2205.1, BIC = 2311.2, model 3 vs. model 2: *LR* = 113.99, *df* = 1, *p* < 0.000). Therefore, it was deemed appropriate to include subjects and items as random factors and to perform mixed logit models.

Second, to find the best fitting model, we successively removed from the previous full model the interaction of second order Age × Sex × SES (model 4: AIC = 2201.3, BIC = 2289.8), then the interactions of first order Age × Sex (model 5: AIC = 2196.3, BIC = 2267.1) and Age × SES (model 6: AIC = 2195.1, BIC = 2248.2) without significant losses of the model fit (model 4 vs. model 3: *LR* = 2.24, *df* = 3, *p* = 0.52; model 5 vs. model 4: *LR* = 1.00, *df* = 3, *p* = 0.80; model 6 vs. model 5: *LR* = 4.79, *df* = 3, *p* = 0.19), indicating that the interactions between Age and Sex or SES did not add significant information to the model. Conversely, removing the interaction SES × Sex lead to a marginally significant loss of the model fit (model 7: AIC = 2196.3, BIC = 2243.5, model 7 vs. model 6: *LR* = 3.19, *df* = 1, *p* = 0.07). With regard to AIC and BIC information, model 6 fitted a little better than model 7, but AIC does not penalize the number of parameters as strongly as BIC (AIC6 = 2195.1 vs. AIC7 = 2196.3). Model 6 was only slightly less parsimonious than model 7 (BIC6 = 2248.2 vs. BIC7 = 2243.5). We therefore chose model 6 based on the LRT results indicating that the interaction between SES and Sex contributed substantial information to the model.

As the main effect of Sex was non-significant (model 6, estimate = -0.17, *SE* = 0.30, *Z* = -0.55, *p* = 0.58) but in the expected direction, we set this main effect at zero (Gelman and Hill, 2007, p. 69) which made it possible to decompose the remaining interaction effect between Sex and SES.

Therefore, we present below model 6 that includes children’s Age, Sex (fixed at 0) and SES as fixed factors and the interaction decomposed between SES and Sex with both subjects and items as random factors. This allowed us to address our first main question, that is, whether sex differences are observed in both high- and low-SES groups. These analyses were completed by running separate models for each Sex group to address our second main question concerning the impact of family SES on boys and girls, in particular whether SES affects only boys or affects both sexes but with a stronger effect for boys.

Average scores of obligatory liaisons correctly produced by children in relation to their socio-demographic characteristics (age group, SES, and sex) are shown in **Table 2**.

**Table 2:** Obligatory liaisons correctly produced by girls and boys in relation to SES and preschool age.

	2–3 years		3–4 years		4–5 years		5–6 years	
	*M*	*SE*	*M*	*SE*	*M*	*SE*	*M*	*SE*
High-SES								
Girls	67.7	6.5	83.3	5.6	86.6	4.4	97.0	1.2
Boys	67.5	6.6	75.1	6.0	85.9	4.1	97.3	1.5
Low-SES								
Girls	47.1	5.8	70.4	5.3	79.2	4.8	92.1	3.3
Boys	28.0	5.4	58.3	7.2	72.4	5.9	78.7	4.4

*Summary of descriptive statistics: M, Mean percentages of correct liaisons over children’s effective responses; SE, standard errors.*

The estimated fixed effects in the binary mixed logit model are summarized in **Table 3**.

**Table 3:** Summary of the fixed effects in the binary mixed model fit by the maximum likelihood.

	Estimate	*SE*	*Z*	*p*
Intercept	0.96	0.34	2.81	0.005
Age = 3–4 years (vs. Age = 2–3 years)	1.17	0.29	4.08	<0.000
Age = 4–5 years (vs. Age = 2–3 years)	1.92	0.29	6.64	<0.000
Age = 5–6 years (vs. Age = 2–3 years)	3.25	0.33	9.94	<0.000
SES = low (vs. SES = high)	-1.05	0.30	-3.51	0.0004
SES = high and male (vs. Female)	-0.16	0.30	-0.54	0.58
SES = low and male (vs. Female)	-0.91	0.29	-3.16	0.002

*N observations = 2691, log-likelihood = -1088.6.*

The GLMM yielded a main effect of Age (all *p* < 0.001, **Table 3**): the productions of correct liaisons by all the children improved significantly with age. To illustrate this notable mastery of obligatory liaisons across the preschool years, the coefficient associated with Age indicates that the log-odds of a correct liaison for 5–6 year-olds were 3.25 log-odds higher than for children in the youngest age group, namely that the odds of a correct liaison for 5–6 year-olds were 25.8 (i.e., e^3.25^) times higher than the odds for 2–3 year-olds. The increase was also significant between the successive later age groups (Tukey *post hoc* comparisons, 4–5 years – 3–4 years: estimate = 0.75, *SE* = 0.28, *Z* = 2.63, *p* = 0.04; 5–6 years – 4–5 years: estimate = 1.33, *SE* = 0.31, *Z* = 4.29, *p* < 0.001) indicating a progressive improvement of correct productions across preschool ages.

Although the children, whatever their sociodemographic characteristics, all mastered progressively the correct production of obligatory liaisons tending with age toward the adults’ categorical use of this type of liaison, the GLMM yielded a main effect of family SES (*p* < 0.001, **Table 3**) with a significant impeding effect of low SES. The odds of a correct liaison for low-SES children were 0.35 (≈e^-1.05^) times lower than the odds for high-SES children.

**FIGURE 1:**
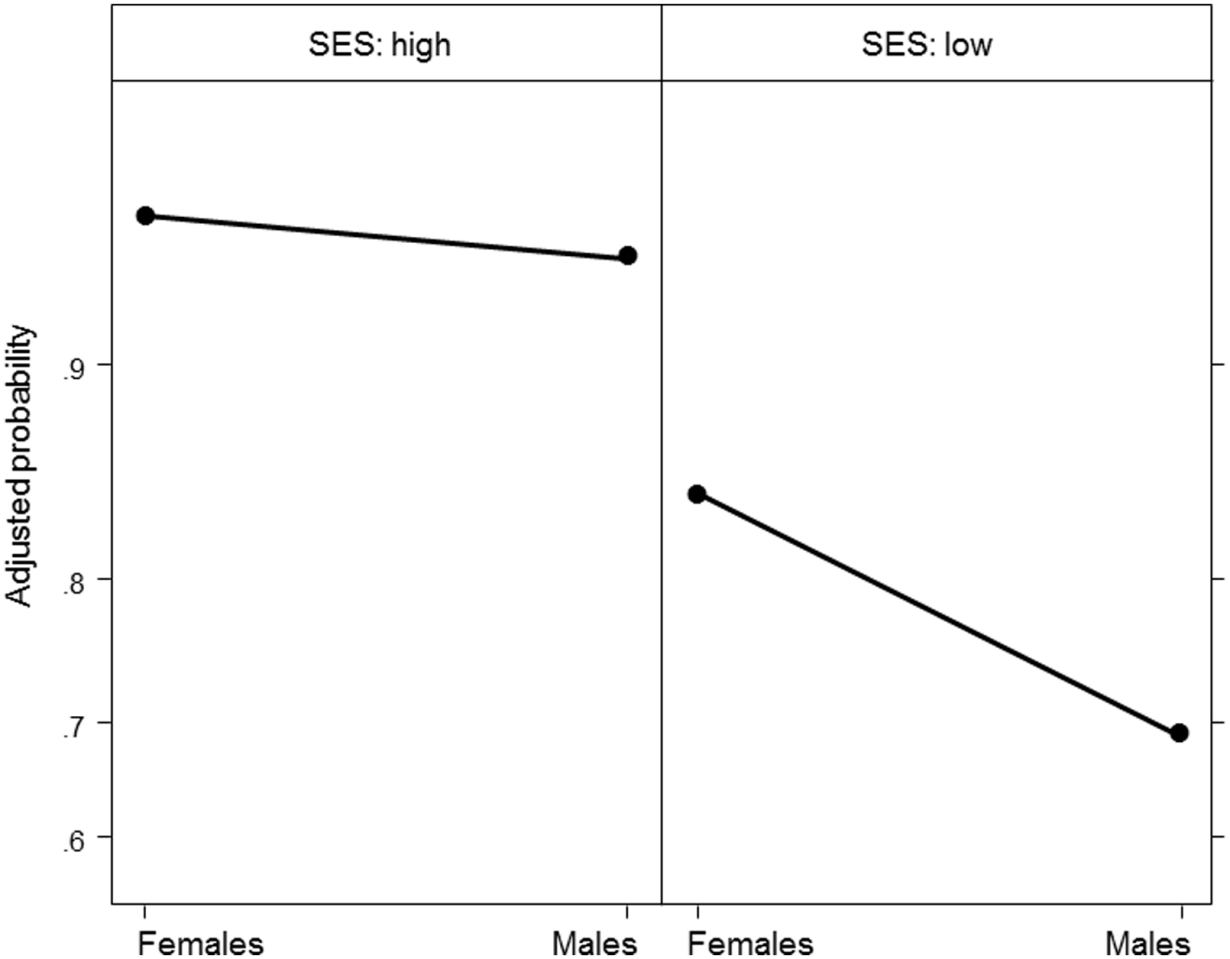
**Adjusted probabilities of obligatory liaisons produced correctly by girls and boys in relation to family socioeconomic status (SES)**.

The step by step selection of the model revealed that child sex alone had no significant effect on children’s correct productions of obligatory liaisons, but instead the GLMM yielded a significant effect of child sex for low-SES children (*p* = 0.002, **Table 3**) but not for high-SES children (*p* = 0.58) as high-SES girls’ and boys’ performances were very similar. Thus, sex differences were not observed across the socioeconomic strata (**Figure 1**). In particular, the analyses indicated that being a low-SES boy impeded the production of correct liaisons: the odds of a correct liaison for low-SES boys were 0.40 (≈e^-0.91^) times lower than the odds for low-SES girls. Moreover, analyzing the effect of family SES for each sex group separately showed that SES had a significant effect for children of both sexes (boys: estimate = -1.77, *SE* = 0.31, *Z* = -5.78, *p* < 0.000; girls: estimate = -1.08, *SE* = 0.30, *Z* = -3.57, *p* = 0.0004).

Finally, *post hoc* comparisons indicated that the performances of low-SES boys were the poorest, significantly lower than those of high-SES children of both sexes (both *p* < 0.001, see **Table 4** for details of the multiple comparisons with Tukey *post hoc* comparisons, **Figure 1**). The performances of low-SES girls were intermediate, as they were significantly lower than those of high-SES children of both sexes (vs. high-SES girls: *p* = 0.003, vs. high-SES boys: *p* = 0.02), but higher than those of low-SES boys (*p* = 0.01).

**Table 4:** Summary of the *post hoc* multiple comparisons between girls and boys from high- and low-SES.

	Estimate	*SE*	*Z*	*p*
Low-SES girls – high-SES girls	-1.05	0.30	-3.53	0.003
High-SES boys – high-SES girls	-0.18	0.30	-0.58	0.94
Low-SES boys – high-SES girls	-1.96	0.30	-6.46	<0.001
High-SES boys – low-SES girls	0.88	0.29	2.98	0.02
Low-SES boys – low-SES girls	-0.90	0.29	-3.13	0.01
Low-SES boys – high-SES boys	-1.78	0.30	-5.96	<0.001

*Tukey *post hoc* comparisons.*

## Discussion

By comparing liaison acquisition between children at the two extremities of the socioeconomic strata across the preschool years, we highlighted that, although correct production of obligatory liaisons progressed with age for all the children, low-SES had a significant impeding effect for children of both sexes, especially for boys. Low-SES boys showed the poorest performances over the preschool years whereas low-SES girls had in-between performances, lower than those of high-SES children of both sexes but higher than those of low-SES boys. Our findings show that child sex alone does not contribute significantly to children’s mastery of obligatory liaisons, but needs to be considered in relation to SES: sex differences were found between low-SES children, but not between high-SES children whose performances were very similar. The interaction between child sex and SES was, however, only marginally significant underlying the fact that family SES nonetheless impacts children’s of both sexes, even if to a greater extent boys.

It must be also noticed that these inter-individual differences in obligatory liaison acquisition are transitory contrary to what has been observed in other aspects of language, such as vocabulary in particular. Indeed, in a previous study, we showed that the magnitude of socioeconomic differences in the production of obligatory liaison decreased with age: it was larger in the first age group (2–3 year-olds) than in the following ones (Barbu et al., 2013). In the present study, we found a major effect of age with an increased mastery of obligatory liaisons throughout the preschool years whatever the children’s sociodemographic characteristics. At the end of the preschool years, all the children progressed toward the adult categorical use of this type of liaisons. This developmental trend and the range of children’s average performances recorded (around 90% of correct liaisons for 5–6 year-olds in the context of determiner + noun) are in line with previous reports on obligatory liaison acquisition by French-speaking children, both for similar experimental studies eliciting the production of obligatory liaisons with picture naming tasks (Chevrot et al., 2009; Dugua et al., 2009) and for corpus-based studies of parent–child’s spontaneous conversations at home (Chevrot et al., 2007).

Our findings enhance understanding of liaison acquisition. In the field of linguistics, liaison is a recurrent issue in adult phonology. In comparison, developmental aspects of liaison have long been neglected and liaison acquisition is a more recent field of investigation (Chevrot et al., 2005, 2009). After recent advances on a developmental scenario for obligatory liaison acquisition (Chevrot et al., 2009; Dugua et al., 2009), studies are still in the early stages when it comes to understanding factors leading to inter-individual variations by showing the influence of family SES (Chevrot et al., 2011; Barbu et al., 2013). Our study is the first to provide evidence of an effect of child sex on liaison acquisition. Moreover, we have shown that the effect of child sex needs to be considered in relation to family SES, and not alone, in order to provide a more accurate picture of the influence of SES on liaison acquisition. This knowledge of inter-individual variations is important for developing our understanding of the processes underpinning liaison acquisition.

In the field of adult phonology, modeling liaison is a challenging issue because its functioning involves different linguistic levels – phonology, lexicon, morphology, and syntax – as well as non-linguistic factors (Chevrot et al., 2005). More generally, this empirical richness makes it an interesting issue for the study of language acquisition, for three reasons: liaison is a strong indicator of frequency effect; liaison errors act as an indicator of a child’s attempt to segment speech into words; and liaison reveals interactions between different levels of linguistic knowledge (Chevrot et al., 2009). Not surprisingly, preliminary results from longitudinal case studies suggest that the acquisition of obligatory liaisons is linked to global indices of language development, such as mean length of utterances or lexical diversity (Liégeois, 2014). These specificities of liaison suggest that the detrimental effect of SES on boys’ performances could be generalized to other aspects of language development.

With regard to inter-individual differences in language development, our findings are in line with recent studies showing the positive role of maternal education (an index of family SES) on overall language comprehension at 18 and 36 months for children of both sexes, but with a greater impact on boys (Zambrana et al., 2012). Our study provides supplementary information by showing that this differential effect of family SES on girls and boys continues throughout the preschool years. This greater impact of family SES on boys than on girls may explain why sex differences seem to increase with decreasing level of parental SES and are found more consistently in lower-SES children across ages (Locke et al., 2002; Fish and Pinkerman, 2003; Reynolds and Fish, 2010). Indeed, our findings highlight that child sex alone does not contribute significantly to children’s abilities to perform obligatory liaisons, but needs to be considered in relation to SES: sex differences were found in low-SES, but not in high-SES children. This may also explain why sex differences are not consistently found in samples that collapse sociodemographic subgroups or focus on particular socioeconomic subgroups. In particular, a large number of developmental studies rely on “convenience samples” of children from higher-SES families who are more inclined to participate in research, raising methodological issues with regard to the representativeness of the results (Fernald et al., 2013). Our findings thus significantly contribute to the debate on sex differences in language abilities and help, at least in part, to resolve apparent contradictory conclusions drawn from previous studies.

Beyond establishing the mere existence of sex differences, attempts to explain these disparities have elicited much debate concerning their social and biological origins. Considering the effects of child sex in relation to other sociodemographic characteristics such as family SES may also help to understand their causes.

Abundant research on language acquisition points to parental language as one of the influential environmental factors explaining variability among children. Both quantitative differences in the linguistic input to which children are exposed (i.e., sheer amount of talk) and qualitative differences (i.e., characteristics of child-directed-speech) influence language growth (Hoff, 2003; Huttenlocher et al., 2010; Rowe, 2012). The frequency of linguistic forms in a child’s input has been shown to be an important factor in acquisition (Cameron-Faulkner et al., 2003). With regard to liaison acquisition, previous studies have suggested that the more children encounter and memorize word1–word2 sequences with a liaison, the earlier they acquire the linguistic material to abstract and generalize the relation between word1 and a specific liaison consonant (e.g., /n/, /z/, or /t/; Dugua et al., 2009). Our experimental task involved word1–world2 sequences encountered both frequently and infrequently in children’s input. As evidenced by previous studies (Dugua et al., 2009), such experimental design affords us the possibility of assessing not only the children’s ability to memorize and store frequent combinations of words involving a liaison, but also their ability to generalize its functioning to word sequences they have rarely or even never encountered. For obligatory liaisons, the input quality does not vary with parent or even child characteristics, as adults systematically produce this type of liaison whatever their sociodemographic characteristics and the situational context of speech (including the identity of the addressee; Durand and Lyche, 2008). Children from all backgrounds are thus exposed to categorical realizations of these linguistic forms, but the difference in the global quantity of the input they receive (Hart and Risley, 2003) induces SES differences in obligatory liaison acquisition rates (Chevrot et al., 2011; Barbu et al., 2013).

As socialization theorists point out, input quantity could also explain sex differences as parents speak more to girls than to boys (Leaper, 2002). This finding is, however, not consistent (Huttenlocher et al., 1991 see also for a review), at least in part because child age is a key moderator. The largest effect of child sex on parents’ talkativeness was found during infancy and toddler years (meta-analysis: Leaper et al., 1998) when input quantity was also found to be the most influential factor in language acquisition (Rowe, 2012). It is thus important to keep in mind that influential factors vary at different ages during development. Moreover, whereas most studies concern middle-class families (Leaper et al., 1998), the question of a differential treatment of girls and boys across socioeconomic groups remains. SES has profound and pervasive effects on parenting: low-SES parents are more concerned that their children conform to societal expectations, are more directive and less conversational than high-SES parents (Hoff et al., 2002) and this can induce family differences in gender language socialization. Although the influence of family SES on gender socialization has been mentioned repeatedly, it remains largely unexplored (Blakemore et al., 2009; Bornstein, 2013). Reduced sex-related differences in children’s language skills in higher-SES families may thus result from less gender-typed parental representations and practices, an issue that needs to be investigated more systematically in future research.

Nevertheless, sex differences have also been reported in low-income families, especially in high-social-risk families, despite seemingly similar family conditions and early experience for children of both sexes (i.e., cognitive and linguistic stimulation, emotional security and attachment, family stress and coping; Morisset et al., 1995). This suggests a differential effect of the early environment on boys’ and girls’ language development. Boys appear more vulnerable to disruptive events and adverse environments (Morisset et al., 1995; Nagy et al., 2007 for reviews). Conversely, girls show earlier and higher social responsiveness and interest (Blakemore et al., 2009). While social responsiveness in infancy predicts subsequent language growth (Fish and Pinkerman, 2003), girls may benefit more from early language input than boys. Therefore, sex differences observed in the patterns of relations between home environment and children’s language skills may be related to early differences in the socio-emotional responsiveness of boys and girls.

Sex differences at birth (e.g., interest in human face: Connellan et al., 2000; eye contact: Hittleman and Dickes, 1979; imitation: Nagy et al., 2007) and correlations with prenatal exposure to testosterone (e.g., eye contact: Lutchmaya et al., 2002a; vocabulary: Lutchmaya et al., 2002b) strongly suggest that biological factors also play a role, at least in early sex differences. High levels of testosterone appear to be a risk factor delaying boys’ language acquisition (Whitehouse et al., 2012), the effect being mediated by children’s socio-emotional engagement (Farrant et al., 2013). This again suggests a relation between early social responsiveness and subsequent language skills. Prenatal hormones may influence brain development. Sex differences in fetal habituation, the most basic form of learning, have attested to maturational differences in neurological development (Hepper et al., 2012). However, the relationship between prenatal hormones and language no longer remains significant when other predictors such as maternal education are taken into account (Farrant et al., 2013) suggesting more complex interactions between biological and environmental factors.

Both genetic and environmental factors contribute to the development of inter-individual differences in language abilities and delays as evidenced by twin studies. The challenge for future research will be to consider more systematically the fact that the relative contributions of biological and environmental factors change throughout development (Hayiou-Thomas et al., 2012) and that the effects are sex- and domain-specific, namely that the weight and the nature of influential factors also differ between sexes as well as between language domains (Galsworthy et al., 2000; Van Hulle et al., 2004). Early sex differences in socio-emotional responsiveness with a possible biological substrate may be the starting point of snowball effects with bidirectional processes between parents and their child (Song et al., 2014), during which family environment may attenuate or enhance early inter-individual differences. Comparisons of contrasted sociodemographic groups across ages should provide a window onto this complex developmental cascade, the stakes being to consider both sides of the issue: the risk factors for boys and the protective factors for girls.

## Conflict of Interest Statement

The authors declare that the research was conducted in the absence of any commercial or financial relationships that could be construed as a potential conflict of interest.
